# Occupational psychosocial stressors and mental distress among healthcare workers during COVID-19 pandemic

**DOI:** 10.31744/einstein_journal/2021AO6281

**Published:** 2021-10-25

**Authors:** João Silvestre Silva-Junior, Arthur Arantes da Cunha, Daniela Campos de Andrade Lourenção, Silmar Maria da Silva, Renata Flavia Abreu da Silva, Magda Guimarães de Araujo Faria, Vivian Aline Mininel, Mirian Cristina dos Santos Almeida, Patrícia Campos Pavan Baptista, Cristiane Helena Gallasch

**Affiliations:** 1 Centro Universitário São Camilo São PauloSP Brazil Centro Universitário São Camilo, São Paulo, SP, Brazil.; 2 Universidade Federal do Amapá MacapáAP Brazil Universidade Federal do Amapá, Macapá, AP, Brazil.; 3 Universidade de São Paulo São PauloSP Brazil Universidade de São Paulo, São Paulo, SP, Brazil.; 4 Universidade Federal de Minas Gerais Belo HorizonteMG Brazil Universidade Federal de Minas Gerais, Belo Horizonte, MG, Brazil.; 5 Universidade Federal do Estado do Rio de Janeiro Rio de JaneiroRJ Brazil Universidade Federal do Estado do Rio de Janeiro, Rio de Janeiro, RJ, Brazil.; 6 Universidade do Estado do Rio de Janeiro Rio de JaneiroRJ Brazil Universidade do Estado do Rio de Janeiro, Rio de Janeiro, RJ, Brazil.; 7 Universidade Federal de São Carlos São CarlosSP Brazil Universidade Federal de São Carlos, São Carlos, SP, Brazil.; 8 Universidade Federal do Tocantins PalmasTO Brazil Universidade Federal do Tocantins, Palmas, TO, Brazil.

**Keywords:** Coronavirus infections, COVID-19, Health personnel, Mental disorders, Occupational health, Epidemiology

## Abstract

**Objective:**

To analyze the factors associated with mental distress among health workers who cared for patients with a suspected or confirmed diagnosis of coronavirus disease 2019 (COVID-19).

**Methods:**

A cross-sectional analytical study of national scope, carried out between in the second quarter of 2020. A total of 437 health professionals, who filled out an electronic form about sociodemographic data, occupational aspects, psychosocial characteristics of work and mental distress. Multiple logistic regression was performed to analyze the covariables associated with mental distress.

**Results:**

There was a predominance of workers on the nursing team (65.0%), female (71.0%), from Southeastern region of the country (68.6%) and with no morbidities (36.2%). The prevalence of mental distress was 61.6%. Job strain was reported by 24% of participants, and the perception of low support from coworkers was described by 52.9%. The final multiple regression model showed that mental distress was associated with females (odds ratio - OR: 1.93; 95%CI: 1.22-3.07), age up to 40 years (OR: 1.64; 95%CI: 1.07-2.52), weekly working hours equal or over 60 hours (OR: 1.87; 95%CI: 1.15-3.11), job strain (OR: 2.45; 95%CI: 1.41-4.40) and low support from co-workers (OR: 3.47; 95%CI: 2.26-5.38).

**Conclusion:**

Six out of ten participants presented mental distress, which was associated to both individual characteristics and factors related to the work carried out during the pandemic. There is an urgent need to map services that have such characteristics, to outline actions to promote mental health and prevent emotional distress at different levels of health care.

## INTRODUCTION

In March 2020, the World Health Organization (WHO) declared a global state of emergency due to the coronavirus disease 2019 (COVID-19), caused by the severe acute respiratory syndrome coronavirus 2 (SARS-CoV-2), a rapidly spreading etiologic agent that causes severe disease.^(1,2)^ It brought repercussions not only to people affected by the disease, but also to healthcare workers responsible for their care. Contamination and illness of professionals involved in patient care are realities in the pandemic, including the record of 364 deaths of healthcare workers due to this infection up to the 49^th^ epidemiological week of 2020, in Brazil.^(3)^ There is an urgent need to monitor these groups with greater potential for illness, based on the actions of specific sectors, such as the frontline of health care.^(4)^

The patient care services underwent abrupt organizational and environmental changes, culminating in modification of their routines. Work intensification was revealed as one of the main characteristics of this process, especially regarding health care activities at the various levels of care. Overcrowded units, lack of equipment, and beds for hospitalization, are problems in the organization of work that impacted the health of the teams in this pandemic context.^(5,6)^ The psychosocial stressors at work can trigger negative emotions of the frontline professionals working against the epidemic, requiring coping strategies for maintenance of their mental health.^(7)^ Therefore, one should reflect on the different possibilities of illness in healthcare workers, beyond the diagnosis of COVID-19.

In this perspective, the recognition of the state of vulnerability, together with the uncertainties experienced in the pandemic period, can have a negative influence on the well-being of healthcare workers.^(7)^ Those who are in direct and frequent contact with patients with suspected or confirmed infection tend to present with a greater risk of psychological overload,^(8)^ and development of both depressive and anxious conditions.^(7,9,10)^ Brazilian data from the *Instituto Nacional de Seguro Social* (INSS) [National Institute of Social Security] indicate, in recent years, such diagnoses are the most frequent reasons for grant disability benefits due to psychiatric illnesses among workers in Brazil. Thus, the early recognition of signs of psychological distress can help in the direction of adequate support and care, to protect the physical and mental health of this group.^(10)^

## OBJECTIVE

To analyze the factors associated with mental suffering among healthcare workers who were caring for patients with suspected or confirmed diagnosis of COVID-19.

## METHODS

An analytical cross-sectional study, conducted between April and June 2020, contemplating data from the first phase of the research entitled Potentials for Strain and Strengthening of Healthcare Workers Acting in Coronavirus Disease 2019 (COVID-19) Care Settings, developed at the *Faculdade de Enfermagem da Universidade do Estado do Rio de Janeiro* (FE-UERJ) and partner organizations.

It was a convenience sample, considering the limitations of face-to-face access of the research subjects and organizations at the critical moment of increase in cases seen in Brazil. Health professionals from several areas, working at all levels of the frontline of care for patients with COVID-19, were invited. The invitation was made through virtual dissemination, via email and social networks, which included the website address with a form to collect sociodemographic data (sex, age, and region of the country where they lived/worked), occupational data (profession, level of care, complexity of health care, characterization of employment status, weekly work hours, and psychosocial factors of work), and clinical data (clinical morbidities and mental distress).

To evaluate the psychosocial characteristics of work, we used the validated version in Brazilian Portuguese of the Job Stress Scale (JSS),^(11)^ based on the model that discusses the workers´ perceptions about the interface between qualitative and quantitative demands of the job, the decision latitude to perform tasks, and the support offered by supervisors and colleagues.^(12)^ The questionnaire contains 17 items, with four response options, on a Likert scale, to evaluate the three dimensions.^(11)^

In assessing mental distress, we used the version validated for Brazilian Portuguese of the Self-Reporting Questionnaire (SRQ-20), developed by the WHO for screening mental disorders.^(13,14)^ There are 20 questions about symptoms of depression, anxiety, and stress, with a dichotomous response (yes/no). Mental suffering is considered when there are seven or more positive responses, regardless of the participant’s sex.^(15)^

Numerical data were presented by means of descriptive statistics (frequencies, means, and standard deviation), and were categorized for analysis. The categorical independent variables were submitted to the test χ^2^ with the outcome of mental distress. Variables with p≤0.20 were selected for the multiple logistic regression model, built with the inclusion of variables according to the increasing order of p value. The stepwise forward method was used, maintaining in the modeling the variables associated with the outcome (p≤0.05), presenting the odds ratio (OR) of the univariate and multiple regression, as well as the 95% confidence interval (95%CI). Data were tabulated in Microsoft^®^ Excel^®^ spreadsheets for Office 365 MSO, version 16.0.12527.20986, and analyzed with the help of the statistical software R, version 1.2.5033.

The research protocol followed the recommendations of Resolution 510 of 2016 of the National Health Council, in addition to its complementary resolutions, registered in *Plataforma Brasil* under CAAE: 30599420.0.0000.0008, and approved by the National Research Ethics Committee (Conep), under protocol 3.979.223. All participants virtually accessed the Informed Consent Form (ICF) and agreed to take part in the research.

## RESULTS

The group participating in the study comprised 437 healthcare workers. Their characteristics are presented on table 1. Most participants were from the nursing staff (65.0%), with a mean age of 38.4 years (standard deviation - SD±10.0). Most were female (71.0%), living and working in the Southeast region of Brazil (68.6%), and with no morbidities (63.8%). The prevalence of mental distress was 61.6%.

**Table 1:** Distribution of healthcare workers according to sociodemographic, occupational, and clinical characteristics - Brazil, 2020

Variable	n (%)	Mental distress		p value

		Yes (n=269)	No (n=168)	
Sex (n=434)				0.0065*
Male	126 (29.0)	65	61	
Female	308 (71.0)	202	106	
Age group, years (n=437)				0.0043*
20-29	90 (20.6)	57	33	
30-39	161 (36.8)	109	52	
40-49	124 (28.4)	73	51	
50-59	49 (11.2)	28	21	
60 or more	13 (3.0)	2	11	
Region (n=437)				0.50^†^
North	79 (18.1)	46	33	
Northeast	30 (6.9)	17	13	
Midwest	8 (1.8)	7	1	
Southeast	300 (68.6)	185	115	
South	20 (4.6)	14	6	
Occupation (n=437)				0.0612^†^
Nurse	243 (55.6)	154	89	
Nurse Technician/Licensed practical nurse	41 (9.4)	28	13	
Physician	69 (15.8)	37	32	
Physical therapist	22 (5.0)	18	4	
Psychologist	15 (3.4)	6	9	
Others	47 (10.8)	26	21	
Number of organizations in which they work (n=435)				0.9022*
1	265 (60.9)	160	105	
2	129 (29.7)	81	48	
3	24 (5.5)	16	8	
4 or more	17 (3.9)	11	6	
Nature of the organization (n=432)				0.5304*
Only public	303 (70.1)	185	118	
Only private	79 (18.3)	46	33	
Mixed	50 (11.6)	34	16	
Type of employment status (n=435)				0.7518*
Only statutory civil servant	136 (31.3)	78	58	
Only subject to the Consolidated Labor Laws (CLT)	145 (33.3)	93	52	
Only temporary contract	45 (10.3)	29	16	
Statutory and CLT	16 (3.7)	9	7	
Other combinations	93 (21.4)	59	34	
Level of health care (n=428)				0.6323*
Primary	135 (31.5)	83	52	
Secondary	79 (18.5)	46	33	
Tertiary	129 (30.1)	83	46	
Quaternary	20 (4.7)	15	5	
More than one level	65 (15.2)	38	27	
Weekly workload, hours (n=434)				0.0731*
<20	17 (3.9)	10	7	
20-39	88 (20.3)	54	34	
40-59	214 (49.3)	121	93	
60 or more	115 (26.5)	82	33	
Demand-control (n=437)				<0.001*
Active	58 (13.3)	36	22	
Passive	128 (29.3)	79	49	
High strain	104 (23.8)	84	20	
Low strain	147 (33.6)	70	77	
Social support at work (n=437)				<0.001*
High	206 (47.1)	94	112	
Low	231 (52.9)	175	56	
Morbidity (n=437)				0.0279*
Yes	158 (36.2)	108	50	
No	279 (63.8)	161	118	

* χ^2^ test; ^†^ Fisher’s exact test.

Most of them were employed only in the public health network (70.1%), in a single organization (60.9%), with a workload of 40 to 59 hours per week (49.3%), under a labor contract (33.3%), and most often working in Primary Care (31.5%).

As for the psychosocial characteristics of the work, the high job strain, with high level of demands and low control over the work, was reported by 24% of participants. The perception of low support from coworkers was described by the majority (52.9%).

The variables sex, age group, occupation, weekly working hours, morbidities, and psychosocial characteristics of the work, including social support, were selected for logistic regression modeling.

In the multiple model, it was observed female participants had a greater than 93.0% odds of reporting mental suffering than males; and those younger than 40 years had a 64.0% greater odds of mental distress than those aged 40 years or older. Regarding characteristics of the work organization, workloads of 60 hours a week or more increased by 87.0% the odds of the outcome among participants of the group. The odds of mental suffering in the group studied was 2.45 times greater when the work was characterized as highly demanding, and 3.47 times greater when there was a perception of low social support at work (Table 2).

**Table 2:** Univariate and multiple logistic regression to study factors associated with mental distress among healthcare workers – Brazil, 2020

Variable	OR	95%CI	OR	95%CI
Sex				
Male	1.00		1.00	
Female	1.79*	1.17-2.73	1.93*	1.22-3.07
Age, years				
Up to 40	1.57^†^	1.07-2.33	1.64^†^	1.07-2.52
40 or more	1.00		1.00	
Profession				
Nursing team	1.35	0.90-2.02		
Others	1.00			
Weekly workload, hours				
<60	1.00		1.00	
60 or more	1.80^†^	1.14-2.88	1.87^†^	1.15-3.11
Morbidity				
No	1.00			
Yes	1.58^†^	1.05-2.40		
Demand-control model				
Others	1.00		1.00	
High strain	3.36^‡^	2.01-5.86	2.45^†^	1.41-4.40
Social support at work				
High	1.00		1.00	
Low	3.72^‡^	2.49-5.62	3.47^‡^	2.26-5.38

* <0.01; ^†^ <0.05; ^‡^ <0.001.

OR: odds ratio; 95%CI: 95% confidence level.

## DISCUSSION

The prevalence of mental suffering found in the present study is higher than that of other national surveys before the pandemic caused by SARS-CoV-2, which analyzed samples of healthcare workers from different areas, and used the same cut-off point in the SRQ-20, indicating a prevalence variability between 21% and 42.6% of mental distress.^(16-18)^The higher rate of cases found in the present study can be explained by the aspects inherent to the context of the COVID-19 pandemic and its impacts throughout society, particularly among healthcare workers.^(6)^

A Brazilian population-based study with more than 45 thousand individuals conducted during the first half of 2020, found that 40.4% of participants often felt sad or depressed, and 52.6% felt anxious or nervous.^(19)^ As for healthcare workers, in Asia, a systematic review of 13 studies conducted during the pandemic with more than 33 thousand participants measured a combined prevalence of anxiety in 23.2% and depression in 22.8%.^(9)^ Those who were on the frontline and involved in direct care of patients with viral infection were at higher risk of depression, anxiety, insomnia, and stress.^(20)^ Since this Brazilian study used the WHO questionnaire, which addresses less specific psychoemotional signs and symptoms, its result may generate a broader estimate of mental distress, and for this reason, it is indicated for screening in health services.^(14)^

The predominance of female participants in the sample is consistent with global data, indicating a higher frequency of women in the healthcare workforce (70%).^(21)^ Data from the present study demonstrated the odds of mental distress among female Brazilian workers was twice as high as among men. In Asia, female healthcare workers had a higher frequency of depressive (26.9% *versus* 20.3%) and anxious (29.1% *versus* 20.9%) symptoms,^(9)^ in addition to more severe mental disorders.^(20)^ This difference between sexes has several psychosocial explanations related to work context, such as women’s lower pay and occupation of less valued positions within the context of health sector of economy.^(21)^ The issue of the double burden (work-home) may be a relevant factor for this result, considering the risk of increasing the overall workload in this pandemic scenario, with the intensification of work and home tasks.

Participants younger than 40 years had a higher odds of mental distress, a result that is in line with pre-pandemic data.^(22,23)^ In a study conducted in the United Kingdom during the global SARS-CoV-2 outbreak, there was no difference between age groups regarding psychological symptoms,^(10)^ but in previous viral outbreak scenarios, younger workers and those with less experience were more likely to present psychoemotional disorders.^(24)^One hypothesis for this result would be that older workers, and probably those with more years of occupation, developed strategies that allowed less traumatic coping with the personal and professional stressors arising from the COVID-19 setting.

No difference was found in the frequency of mental distress among the occupations of the research participants. However, a systematic review indicated that the nursing team had a higher frequency of depressive and anxious conditions than did the medical staff;^(9)^ and a Chinese study showed more severe symptoms among nursing staff.^(20)^ Perhaps the complexity of the Brazilian pandemic context, with collective stressors of the work environment and organization, promoted an equanimous negative impact among professionals, regardless of the specific issues of each job. For example, the unavailability of personal protection equipment and work overload,^(25)^ in addition to the fear of being contaminated, may be common and frequent psychological stressors among those who are on the frontline of patient care.^(8)^ The qualitative study with nursing professionals in China showed the team exhibited both positive and negative feelings during the pandemic, requiring the development of individual and collective coping strategies to mitigate the negative impacts of work, and to maintain their professional performance.^(7)^

The review by Shaukat et al.,^(6)^ relates mental distress to psychosocial conditions of work of healthcare professionals during this pandemic. The data from the present Brazilian study showed psychosocial work characteristics were significantly associated with mental suffering. The high job strain, with high qualitative and quantitative demand of tasks and low decision latitude to perform them, was not a frequent characteristic among participants, but it increased the odds of illness under study by almost 150%. In Germany, increased workload and organizational changes were found to be associated with burnout and psychological distress among healthcare workers.^(8)^ Other studies conducted with healthcare workers before the pandemic found similar results regarding the association between toxic work stressors and mental distress.^(16,18)^ In addition to the stressors common to work in health services, there are probably characteristics inherent to the Brazilian public health system, such as job strain, reduced availability of supplies, and precarious labor relations, which may have been aggravated by the scenario of fighting COVID-19.^(26)^

Another example of work stressors is the long working day. A weekly workload of 60 hours or more increased the chance of fatigue in the group studied. With an increase in the overall workload, the change in work organization after the onset of the pandemic was associated with depression (OR: 2.00; 95%CI: 1.33-3.02) and anxiety (OR: 2.24; 95%CI: 1.50-3.36) among healthcare workers in China.^(27)^ After all, a longer workday like that of the participants in this study, increases the time in contact with negative aspects at work, deteriorating physical and emotional health.^(28)^

In a scenario of infection risk, management of the effectiveness of actions to protect workers from the biological agent should be permanent.^(6)^In Germany, workers suggested actions to improve working conditions during the pandemic, such as adaptation of teams to the demand for services, clarity in organizational and planning guidelines, and better communication between managers and the team.^(8)^ China, the first country affected by the infection, established guidelines^(29)^ to control the negative impact of the pandemic on workers’ health, such as the balance between working and resting hours, strengthening safety actions, and offering health support.^(28)^

The perception of good relationships among coworkers and managers can influence health positively.^(12)^ This social support can modulate and balance stress-generating contexts, and can cause demands to be experienced as stimuli, resulting in a lower “cost of accomplishment” of the task to be performed.^(30)^ In this sense, as observed in the present study and in other investigations,^(16,18)^ healthcare workers with perception of low social support at work had a greater chance of mental suffering. ^(16,18)^ In the pandemic scenario, with increased physical and emotional burden at work, combined with limited access to psychological support services,^(28)^ the high odds of mental distress with a lower level of support from colleagues, is understandable.^(8,31)^

In this pandemic context, the Chinese recommendations include face-to-face support to deal with the psychological burden and the offer of telehealth services.^(29)^ Therefore, in addition to improving working conditions, there are gains when organizing teams offer shelter, support, and collective and individual interventions to workers.^(6,31)^ Strategies can be developed to minimize fear of the risk of getting sick or infecting acquaintances, living with pain and death of patients and coworkers, and other issues that cause stress, such as the precarious working conditions illustrated in the results of this research. It is important that such actions be implemented in a preventive manner for all workers, and established as a permanent organizational policy of health and safety at work.

Considering that this is a pioneer study in the evaluation of workers’ mental health and psychosocial stressors at work during the pandemic of COVID-19, with validated psychometric scales and statistical control techniques for confounding variables, there are limitations that should be taken into consideration when extrapolating its results. Notably, there was the participation of individuals with better access to technological tools and internet connectivity; despite the national scope, there is a mismatch between the demographic distribution of the country and the place of residence of participants; the greater research interest of people with signs and symptoms of mental distress and the impossibility of assessing losses; the use of self-reported questionnaires, which may be impacted by the cognitive repercussion of the participants’ clinical condition; and the cross-sectional design of the study, which does not allow inferring causality among outcome and covariates.

## CONCLUSION

Mental suffering was present in six out of ten healthcare workers, who participated in the study and were engaged in patient care during the COVID-19 pandemic. Individual factors influenced the increased chance of mental distress, such as female sex and age under 40 years.

Psychosocial characteristics of the job showed a strong association with the outcome, such as perceived high job strain at work, weekly workload of 60 or more hours, and low level of support from coworkers. Thus, there is an urgent need to map health services with these characteristics, to plan actions that promote mental health and prevent emotional distress at the various levels of care.

Considering the impact of the aspects of work context, and the content found in this group, a national policy for the evaluation and mitigation of psychosocial work risk is mandatory. The implementation of strategies that protect healthcare workers from stressful conditions may help to control the permanent negative repercussions on the workers’ emotional well-being and quality of life.

Finally, additional longitudinal design studies with representative samples from each region of the country are suggested, to deepen the national discussion about the impact of work on workers’ health, and to enable indicating effective interventions.

## References

[1] World Health Organization (WHO). Naming the coronavirus disease (COVID-19) and the virus that causes it. Geneva: WHO; 2020 [cited 2020 Nov 11]. Available from: https://www.who.int/emergencies/diseases/novel-coronavirus-2019/technical-guidance/naming-the-coronavirus-disease-(covid-2019)-and-the-virus-that-causes-it

[2] Pan American Health Organization (PAHO). WHO characterizes COVID-19 as a pandemic. Washington (DC): PAHO; 2020 [cited 2020 Nov 11]. Available from: https://www.paho.org/en/news/11-3-2020-who-characterizes-covid-19-pandemic

[3] Brasil. Ministério da Saúde. Secretaria de Vigilância em Saúde. Boletim epidemiológico especial: doença pelo coronavírus COVID-19. Semana epidemiológica 49 (29/11 a 05/12/2020). Brasília (DF): Ministério da Saúde; 2020 [citado 2020 Dez 14]. Disponível em: https://www.gov.br/saude/pt-br/media/pdf/2020/dezembro/11/boletim_epidemiologico_covid_40-1.pdf

[4] Nguyen LH, Drew DA, Graham MS, Joshi AD, Guo CG, Ma W, Mehta RS, Warner ET, Sikavi DR, Lo CH, Kwon S, Song M, Mucci LA, Stampfer MJ, Willett WC, Eliassen AH, Hart JE, Chavarro JE, Rich-Edwards JW, Davies R, Capdevila J, Lee KA, Lochlainn MN, Varsavsky T, Sudre CH, Cardoso MJ, Wolf J, Spector TD, Ourselin S, Steves CJ, Chan AT; Coronavirus Pandemic Epidemiology Consortium. Risk of COVID-19 among front-line health-care workers and the general community: a prospective cohort study. Lancet Public Heal. 2020;5(9):e475-83. 10.1016/S2468-2667(20)30164-X PMC7491202 32745512

[5] Liu Y, Li J, Feng Y. Critical care response to a hospital outbreak of the 2019-nCoV infection in Shenzhen, China. Crit Care. 2020;24(1):56. 10.1186/s13054-020-2786-x PMC7029610 32070391

[6] Shaukat N, Ali DM, Razzak J. Physical and mental health impacts of COVID-19 on healthcare workers: a scoping review. Int J Emerg Med. 2020;13(1):40. Review. 10.1186/s12245-020-00299-5 PMC7370263 32689925

[7] Sun N, Wei L, Shi S, Jiao D, Song R, Ma L, et al. A qualitative study on the psychological experience of caregivers of COVID-19 patients. Am J Infect Control. 2020;48(6):592-8. 10.1016/j.ajic.2020.03.018 PMC7141468 32334904

[8] Zerbini G, Ebigbo A, Reicherts P, Kunz M, Messman H. Psychosocial burden of healthcare professionals in times of COVID-19 - a survey conducted at the University Hospital Augsburg. Ger Med Sci. 2020;18:Doc05. 10.3205/000281 PMC7314868 32595421

[9] Pappa S, Ntella V, Giannakas T, Giannakoulis VG, Papoutsi E, Katsaounou P. Prevalence of depression, anxiety, and insomnia among healthcare workers during the COVID-19 pandemic: a systematic review and meta-analysis. Brain Behav Immun. 2020;88:901-7. Erratum in: Brain Behav Immun. 2021;92:247. 10.1016/j.bbi.2020.05.026 PMC7206431 32437915

[10] Choudhury T, Debski M, Wiper A, Abdelrahman A, Wild S, Chalil S, et al. COVID-19 pandemic: looking after the mental health of our healthcare workers. J Occup Environ Med. 2020;62(7):e373-6. 10.1097/JOM.0000000000001907 32730043

[11] Alves MG, Chor D, Faerstein E, Lopes Cde S, Werneck GL. Short version of the “job stress scale”: a Portuguese-language adaptation. Rev Saude Publica. 2004;38(2):164-71. 10.1590/s0034-89102004000200003 15122370

[12] Araújo TM, Graça CC, Araújo E. Estresse ocupacional e saúde: contribuições do modelo demanda-controle. Cien Saude Colet. 2003;8(4):991-1003.

[13] Mari JJ, Williams P. A validity study of a psychiatric screening questionnaire (SRQ-20) in primary care in the city of Sao Paulo. Br J Psychiatry. 1986;148:23-6. 10.1192/bjp.148.1.23 3955316

[14] World Health Organization (WHO). A user’s guide to the Self Reporting Questionnaire (SRQ). Geneva: WHO; 1994 [cited 2020 Nov 11]. Available from: https://apps.who.int/iris/bitstream/handle/10665/61113/WHO_MNH_PSF_94.8.pdf?sequence=1&isAllowed=y

[15] Gonçalves DM, Stein AT, Kapczinski F. Avaliação de desempenho do Self-Reporting Questionnaire como instrumento de rastreamento psiquiátrico: um estudo comparativo com o Structured Clinical Interview for DSM-IV-TR. Cad Saude Publica. 2008;24(2):380-90. 10.1590/s0102-311x2008000200017 18278285

[16] Araújo TM, Mattos AI, Almeida MM, Santos KO. Psychosocial aspects of work and common mental disorders among health workers: contributions of combined models. Rev Bras Epidemiol. 2016;19(3):645-57. 10.1590/1980-5497201600030014 27849277

[17] Braga LC, Carvalho LR, Binder MC. Condições de trabalho e transtornos mentais comuns em trabalhadores da rede básica de saúde de Botucatu (SP). Cien Saude Colet. 2010;15(Suppl 1):1585-96. 10.1590/s1413-81232010000700070 20640320

[18] Carvalho DB, Araújo TM, Bernardes KO. Transtornos mentais comuns em trabalhadores da Atenção Básica à Saúde. Rev Bras Saúde Ocup. 2016;41:e17.

[19] Barros MB, Lima MG, Malta DC, Szwarcwald CL, Azevedo RC, Romero D, et al. Report on sadness/depression, nervousness/anxiety and sleep problems in the Brazilian adult population during the COVID-19 pandemic. Epidemiol Serv Saude. 2020;29(4):e2020427. 10.1590/s1679-49742020000400018 32844918

[20] Lai J, Ma S, Wang Y, Cai Z, Hu J, Wei N, et al. Factors associated with mental health outcomes among health care workers exposed to Coronavirus Disease 2019. JAMA Netw Open. 2020;3(3):e203976. 10.1001/jamanetworkopen.2020.3976 PMC7090843 32202646

[21] Boniol M, McIsaac M, Xu L, Wuliji T, Diallo K, Campbell J. Gender equity in the health workforce: analysis of 104 countries. Health Workforce Working paper 1. March 2019. Geneva: World Health Organization; 2019 [cited 2020 Nov 11]. Available from: https://apps.who.int/iris/bitstream/handle/10665/311314/WHO-HIS-HWF-Gender-WP1-2019.1-eng.pdf?ua=1

[22] Nascimento Sobrinho CL, Carvalho FM, Bonfim TA, Cirino CA, Ferreira IS. Condições de trabalho e saúde mental dos médicos de Salvador, Bahia, Brasil. Cad Saude Publica. 2006;22(1):131-40. 10.1590/s0102-311x2006000100014 16470290

[23] Dilélio AS, Facchini LA, Tomasi E, Silva SM, Thumé E, Piccini RX, et al. Prevalência de transtornos psiquiátricos menores em trabalhadores da atenção primária à saúde das regiões Sul e Nordeste do Brasil. Cad Saude Publica. 2012;28(3):503-14. 10.1590/s0102-311x2012000300011 22415183

[24] Kisely S, Warren N, McMahon L, Dalais C, Henry I, Siskind D. Occurrence, prevention, and management of the psychological effects of emerging virus outbreaks on healthcare workers: rapid review and meta-analysis. BMJ. 2020;369:m1642. Review. 10.1136/bmj.m1642 PMC7199468 32371466

[25] Mhango M, Dzobo M, Chitungo I, Dzinamarira T. COVID-19 risk factors among health workers: a rapid review. Saf Health Work. 2020;11(3):262-5. Review. 10.1016/j.shaw.2020.06.001 PMC7502606 32995051

[26] Teixeira CF, Soares CM, Souza EA, Lisboa ES, Pinto IC, Andrade LR, et al. The health of healthcare professional coping with the Covid-19 pandemic. Cien Saude Colet. 2020;25(9):3465-74. 10.1590/1413-81232020259.19562020 32876270

[27] Chen J, Liu X, Wang D, Jin Y, He M, Ma Y, et al. Risk factors for depression and anxiety in healthcare workers deployed during the COVID-19 outbreak in China. Soc Psychiatry Psychiatr Epidemiol. 2021;56(1):47-55. 10.1007/s00127-020-01954-1 PMC7483060 32914298

[28] Ayanian JZ. Mental health needs of health care workers providing frontline COVID-19 care. JAMA Health Forum. 2020;1(4):e200397. 10.1001/jamahealthforum.2020.0397 36218605

[29] Zhou Y, Zhou Y, Song Y, Ren L, Ng CH, Xiang YT, et al. Tackling the mental health burden of frontline healthcare staff in the COVID-19 pandemic: China’s experiences. Psychol Med. 2021;51(11):1955-6. 10.1017/S0033291720001622 PMC7417977 32398180

[30] Frankenhauser M. A biopsychosocial approach to work life issues. Int J Health Serv. 1989;19(4):747-58. Review. 10.2190/01DY-UD40-10M3-CKY4 2684874

[31] Xiao H, Zhang Y, Kong D, Li S, Yang N. The effects of social support on sleep quality of medical staff treating patients with Coronavirus Disease 2019 (COVID-19) in January and February 2020 in China. Med Sci Monit. 2020;26:e923549. 10.12659/MSM.923549 PMC7075079 32132521

