# Supplementary material for: Occupational psychosocial stressors and mental distress among healthcare workers during COVID-19 pandemic
Source: Einstein (Sao Paulo). 2021 Oct 25;19:eAO6281. doi: 10.31744/einstein_journal/2021AO6281 (PMC8555877; doi:10.31744/einstein_journal/2021AO6281)
Supplement: Translation

# Translation: Estressores psicossociais ocupacionais e sofrimento mental em trabalhadores de saúde na pandemia de COVID-19

**Authors:** João Silvestre Silva-Junior, Arthur Arantes da Cunha, Daniela Campos de Andrade Lourenção, Silmar Maria da Silva, Renata Flavia Abreu da Silva, Magda Guimarães de Araujo Faria, Vivian Aline Mininel, Mirian Cristina dos Santos Almeida, Patrícia Campos Pavan Baptista, Cristiane Helena Gallasch

**Affiliations:** 1 Brasil Centro Universitário São Camilo, São Paulo, SP, Brasil.; 2 Brasil Universidade Federal do Amapá, Macapá, AP, Brasil.; 3 Brasil Universidade de São Paulo, São Paulo, SP, Brasil.; 4 Brasil Universidade Federal de Minas Gerais, Belo Horizonte, MG, Brasil.; 5 Brasil Universidade Federal do Estado do Rio de Janeiro, Rio de Janeiro, RJ, Brasil.; 6 Brasil Universidade do Estado do Rio de Janeiro, Rio de Janeiro, RJ, Brasil.; 7 Brasil Universidade Federal de São Carlos, São Carlos, SP, Brasil.; 8 Brasil Universidade Federal do Tocantins, Palmas, TO, Brasil.

**Keywords:** Infecções por coronavírus, COVID-19, Pessoal de saúde, Transtornos mentais, Saúde do trabalhador, Epidemiologia

## Abstract

**Objetivo:**

Analisar os fatores associados ao sofrimento mental de trabalhadores de saúde que atuavam na assistência a pacientes com diagnóstico suspeito ou confirmado de doença pelo coronavírus 2019 (COVID-19).

**Métodos:**

Estudo transversal analítico de abrangência nacional, realizado no segundo trimestre de 2020. Participaram 437 profissionais de saúde que preencheram formulário eletrônico sobre dados sociodemográficos, aspectos ocupacionais, características psicossociais do trabalho e sofrimento mental. Foi realizada regressão logística múltipla para analisar as covariáveis associadas ao sofrimento mental.

**Resultados:**

Predominaram trabalhadores da equipe de enfermagem (65,0%), do sexo feminino (71,0%), da região Sudeste do país (68,6%) e sem morbidades (36,2%). A prevalência de sofrimento mental foi de 61,6%. O trabalho de alta exigência psicossocial foi informado por 24% dos participantes e a percepção de baixo apoio dos colegas de trabalho foi relatada por 52,9%. O modelo de regressão múltiplo final demonstrou que o sofrimento mental estava associado a: sexo feminino (razão de chance - RC: 1,93; IC95%: 1,22-3,07), idade até 40 anos (RC: 1,64; IC95%: 1,07-2,52), jornada semanal de trabalho igual ou superior a 60 horas (RC: 1,87; IC95%: 1,15-3,11), trabalho de alta exigência (RC: 2,45; IC95%: 1,41-4,40) e baixo apoio dos colegas (RC: 3,47; IC95%: 2,26-5,38).

**Conclusão:**

Seis em cada dez participantes apresentavam quadro de sofrimento mental associado tanto a características individuais, quanto a fatores relacionados ao trabalho realizado durante a pandemia. É urgente a necessidade de mapear os serviços que tenham tais características, para delinear ações de promoção da saúde mental e prevenção do desgaste emocional nos diversos níveis de atenção em saúde.

## INTRODUÇÃO

O estado de emergência global decretado pela Organização Mundial da Saúde (OMS), em março de 2020, devido à doença pelo coronavírus 2019 (COVID-19) causada pelo coronavírus da síndrome respiratória aguda grave 2 (SARS-CoV-2), um agente etiológico de rápida propagação e causador de grave doença,^(1,2)^ trouxe repercussões não somente às pessoas afetadas pela doença, mas também aos trabalhadores da saúde, responsáveis por essa assistência. A contaminação e o adoecimento dos profissionais envolvidos no atendimento dos pacientes são realidades na pandemia, inclusive com o registro de 364 casos de óbito de trabalhadores da saúde por essa infecção até a 49ª semana epidemiológica de 2020 no Brasil.^(3)^ Nesse sentido, urge a necessidade de acompanhamento desses grupos com maior potencial de adoecimento, a partir da atuação em setores específicos, como a linha de frente de cuidado com a saúde.^(4)^

Os serviços de atendimento dos pacientes sofreram mudanças organizacionais e ambientais abruptas, culminando na alteração de suas rotinas. A intensificação do trabalho foi desvelada como uma das principais características desse processo, em especial quanto às atividades de assistência à saúde, nos diversos níveis de atenção. A superlotação das unidades, a falta de equipamentos para cuidados e de leitos para internação são problemas na organização do trabalho que impactaram na saúde das equipes, nesse contexto pandêmico.^(5,6)^ Os estressores psicossociais no trabalho podem desencadear emoções negativas dos profissionais de linha de frente contra a epidemia, requerendo estratégias de enfrentamento para a manutenção de sua saúde mental.^(7)^ Deve-se, então, refletir sobre as diferentes possibilidades de adoecimento dos trabalhadores de saúde, para além do diagnóstico de COVID-19.

Nessa perspectiva, o reconhecimento do estado de vulnerabilidade, aliado às incertezas vivenciadas no período pandêmico, pode exercer influência negativa no bem-estar dos profissionais de saúde.^(7)^ Aqueles que estão em contato direto e frequente com pacientes com suspeita ou confirmação de infecção tendem a apresentar maior risco de sobrecarga psicológica,^(8)^ e desenvolvimento tanto de quadros depressivos, quanto ansiosos.^(7,9,10)^ Dados do Instituto Nacional do Seguro Social (INSS) indicam que nos últimos anos tais diagnósticos são os motivos mais frequentes de incapacidade laborativa por doenças psiquiátrica entre trabalhadores no Brasil. Desse modo, o reconhecimento precoce de sinais de sofrimento psicológico pode auxiliar no direcionamento para suporte e cuidados adequados, a fim de proteger a saúde física e mental desse grupo.^(10)^

## OBJETIVO

Analisar os fatores associados ao sofrimento mental de trabalhadores de saúde que atuavam na assistência a pacientes com diagnóstico suspeito ou confirmado de COVID-19.

## MÉTODOS

Estudo transversal analítico, realizado entre abril e junho de 2020, contemplando dados da primeira fase da pesquisa intitulada Potenciais de Desgaste e Fortalecimento dos Trabalhadores de Saúde Atuantes nos Cenários de Atendimento à Doença por Coronavírus 2019 (COVID-19), desenvolvido na Faculdade de Enfermagem da Universidade do Estado do Rio de Janeiro (FE-UERJ) e instituições parceiras.

A amostra foi composta por conveniência, considerando-se as limitações de acesso presencial dos sujeitos de pesquisa e das instituições no momento crítico do aumento de casos atendidos no Brasil. Foram convidados profissionais de saúde de diversas áreas, atuantes em todos os níveis de atenção, na linha de frente do cuidado com pacientes com COVID-19. O convite ocorreu por divulgação virtual, via *e-mail* e redes sociais, que incluiu o endereço da página eletrônica com formulário para coleta de dados sociodemográficos (sexo, idade e região do país onde residia/trabalhava), ocupacionais (profissão, nível de atenção, complexidade de assistência à saúde, caracterização de vínculo institucional, jornada semanal de trabalho e fatores psicossociais do trabalho) e clínicos (morbidades clínicas e sofrimento mental).

Para avaliar as características psicossociais do trabalho, foi utilizada a versão validada para o português da *Job Stress Scale* (JSS),^(11)^ baseada no modelo que discute a percepção do trabalhador quanto à interface entre as demandas qualiquantitativas do trabalho, o controle/a latitude de decisão para cumprimento das tarefas e o apoio oferecido por supervisores e colegas.^(12)^ O questionário contém 17 itens, com quatro opções de resposta em escala do tipo Likert para avaliar as três dimensões.^(11)^

Na avaliação do sofrimento mental, foi utilizada a versão validada para o português falado no Brasil do *Self-Reporting Questionnaire* (SRQ-20), desenvolvido pela *World Health Organization* (WHO) para fins de triagem de transtornos mentais.^(13,14)^ São 20 perguntas sobre sintomas de depressão, ansiedade e estresse, com resposta dicotômica (sim/não), sendo considerado quadro de sofrimento mental quando há sete ou mais respostas positivas, independentemente do sexo do participante.^(15)^

Os dados numéricos foram apresentados por meio de estatística descritiva (frequências, médias e desvio padrão) e foram categorizadas para análise. As variáveis independentes categóricas foram submetidas ao teste do χ^2^ com o desfecho sofrimento mental. As variáveis cujo valor de p fossem iguais ou menores que 0,20 foram selecionadas para o modelo de regressão logística múltiplo, construído com inclusão das variáveis conforme a ordem crescente do valor de p. Foi utilizado o método de *stepwise forward* mantendo na modelagem as variáveis associadas ao desfecho (p≤0,05), apresentando razão de chance (RC) da regressão univariada e múltipla, assim como o intervalo de confiança de 95% (IC95%). Os dados foram tabulados em planilhas do Microsoft^®^ Excel^®^ para Office 365 MSO, versão 16.0.12527.20986, e analisados com auxílio do software estatístico R, versão 1.2.5033.

O protocolo de pesquisa seguiu as recomendações da resolução 510 de 2016 do Conselho Nacional de Saúde, além de suas resoluções complementares, sendo registrado na Plataforma Brasil sob o CAAE: 30599420.0.0000.0008 e aprovado pela Comissão Nacional de Ética em Pesquisa (Conep), sob parecer 3.979.223. Todos os participantes acessaram virtualmente o Termo de Consentimento Livre e Esclarecido (TCLE) e aceitaram fazer parte da pesquisa.

## RESULTADOS

O grupo que participou do estudo foi de 437 profissionais da saúde. Suas características são apresentadas na tabela 1. A maioria de participantes foi da equipe de enfermagem (65,0%), com média de idade de 38,4 anos (desvio-padrão - DP±10,0). O perfil da maioria dos participantes era sexo feminino (71,0%), morando e trabalhando na região Sudeste do país (68,6%) e sem morbidades (63,8%). A prevalência de sofrimento mental foi de 61,6%.

**Tabela 1:** Distribuição dos trabalhadores de serviços de saúde conforme características sociodemográficas, ocupacionais e clínicas – Brasil, 2020

Variável	n (%)	Sofrimento mental		Valor de p

		Sim (n=269)	Não (n=168)	
Sexo (n=434)				0,0065*
Masculino	126 (29,0)	65	61	
Feminino	308 (71,0)	202	106	
Faixa etária, anos (n=437)				0,0043*
20-29	90 (20,6)	57	33	
30-39	161 (36,8)	109	52	
40-49	124 (28,4)	73	51	
50-59	49 (11,2)	28	21	
60 ou mais	13 (3,0)	2	11	
Região (n=437)				0,50^†^
Norte	79 (18,1)	46	33	
Nordeste	30 (6,9)	17	13	
Centro-Oeste	8 (1,8)	7	1	
Sudeste	300 (68,6)	185	115	
Sul	20 (4,6)	14	6	
Profissão (n=437)				0,0612^†^
Enfermeiros	243 (55,6)	154	89	
Técnico/auxiliar de enfermagem	41 (9,4)	28	13	
Médico	69 (15,8)	37	32	
Fisioterapeuta	22 (5,0)	18	4	
Psicólogo	15 (3,4)	6	9	
Outros	47 (10,8)	26	21	
Número de instituições em que trabalha (n=435)				0,9022*
1	265 (60,9)	160	105	
2	129 (29,7)	81	48	
3	24 (5,5)	16	8	
4 ou mais	17 (3,9)	11	6	
Natureza da instituição (n=432)				0,5304*
Apenas pública	303 (70,1)	185	118	
Apenas privada	79 (18,3)	46	33	
Mista	50 (11,6)	34	16	
Tipo de vínculo (n=435)				0,7518*
Apenas estatutário	136 (31,3)	78	58	
Apenas celetista	145 (33,3)	93	52	
Apenas contrato temporário	45 (10,3)	29	16	
Estatutário e celetista	16 (3,7)	9	7	
Outras combinações	93 (21,4)	59	34	
Nível de assistência em saúde (n=428)				0,6323*
Primário	135 (31,5)	83	52	
Secundário	79 (18,5)	46	33	
Terciário	129 (30,1)	83	46	
Quaternário	20 (4,7)	15	5	
Mais de um nível	65 (15,2)	38	27	
Jornada semanal, horas (n=434)				0,0731*
<20	17 (3,9)	10	7	
20-39	88 (20,3)	54	34	
40-59	214 (49,3)	121	93	
60 ou mais	115 (26,5)	82	33	
Demanda-controle (n=437)				<0,001*
Trabalho ativo	58 (13,3)	36	22	
Trabalho passivo	128 (29,3)	79	49	
Alta exigência	104 (23,8)	84	20	
Baixa exigência	147 (33,6)	70	77	
Apoio no trabalho (n=437)				<0,001*
Alto	206 (47,1)	94	112	
Baixo	231 (52,9)	175	56	
Morbidade (n=437)				0,0279*
Sim	158 (36,2)	108	50	
Não	279 (63,8)	161	118	

* teste do χ^2^; ^†^ teste exato de Fisher.

A maioria possuía vínculo apenas na rede pública de saúde (70,1%), em uma única instituição (60,9%), com carga horária de 40 a 59 horas semanais (49,3%), regime contratual celetista (33,3%) e atuação mais frequente na Atenção Primária (31,5%).

Quanto às características psicossociais do trabalho, a alta exigência (*job strain*), com alto nível de demandas e baixo controle sobre o trabalho, foi informada por 24% dos participantes. A percepção de baixo apoio dos colegas de trabalho foi maioria (52,9%).

Foram selecionadas para modelagem da regressão logística as variáveis sexo, faixa etária, profissão, jornada semanal de trabalho, morbidades e características psicossociais do trabalho, incluindo apoio social.

No modelo múltiplo, foi observado que participantes do sexo feminino tiveram chance maior que 93,0% de relatar sofrimento mental, comparando com o sexo masculino; e aqueles de faixa etária inferior a 40 anos tiveram chance 64,0% maior de sofrimento mental, do que aqueles com 40 ou mais anos. Quanto às características da organização do trabalho, a jornada igual ou superior a 60 horas semanais aumentou em 87,0% a chance do desfecho entre os participantes do grupo. A chance do sofrimento mental no grupo estudado foi 2,45 vezes maior quando o trabalho foi caracterizado como de alta exigência, e 3,47 vezes maior quando houve percepção de baixo apoio social no trabalho (Tabela 2).

**Tabela 2:** Regressão logística univariada e múltipla para estudo dos fatores associados ao sofrimento mental entre profissionais de saúde – Brasil, 2020

Variável	RC	IC95%	RC	IC95%
Sexo				
Masculino	1,00		1,00	
Feminino	1,79*	1,17-2,73	1,93*	1,22-3,07
Idade, anos				
Até 40	1,57^†^	1,07-2,33	1,64^†^	1,07-2,52
40 ou mais	1,00		1,00	
Profissão				
Equipe enfermagem	1,35	0,90-2,02		
Outros	1,00			
Jornada semanal, horas				
<60	1,00		1,00	
60 ou mais	1,80^†^	1,14-2,88	1,87^†^	1,15-3,11
Morbidade				
Não	1,00			
Sim	1,58^†^	1,05-2,40		
Modelo demanda-controle				
Outros	1,00		1,00	
Alta exigência	3,36^‡^	2,01-5,86	2,45^†^	1,41-4,40
Apoio social no trabalho				
Alto	1,00		1,00	
Baixo	3,72^‡^	2,49-5,62	3,47^‡^	2,26-5,38

* <0,01; ^†^ <0,05; ^‡^ <0,001.

RC: razão de chances; IC95%: intervalo de confiança de 95%.

## DISCUSSÃO

A prevalência de sofrimento mental encontrada no presente estudo é superior à de outras pesquisas nacionais anteriores à pandemia causada pelo SARS-CoV-2, que analisaram amostras de profissionais da saúde de diferentes áreas e utilizaram o mesmo ponto de corte no SRQ-20, as quais indicaram variabilidade de prevalência entre 21% e 42,6% de sofrimento mental.^(16-18)^ A taxa mais elevada de casos encontrada no presente estudo pode ser explicada pelos aspectos inerentes ao contexto da pandemia de COVID-19 e por seus impactos em toda a sociedade e, particularmente, entre os trabalhadores da área da saúde.^(6)^

Estudo brasileiro de base populacional com mais de 45 mil participantes realizado no primeiro semestre de 2020 verificou que 40,4% dos participantes frequentemente se sentiam tristes ou deprimidos, e 52,6% se sentiam ansiosos ou nervosos.^(19)^ Quanto aos trabalhadores da saúde, revisão sistemática de 13 estudos realizados durante a pandemia somando mais de 33 mil participantes na Ásia mensurou prevalência combinada de ansiedade em 23,2% e depressão em 22,8%.^(9)^ Aqueles que estavam na linha de frente e envolvidos no atendimento direto do paciente com a infecção viral apresentavam maior risco de depressão, ansiedade, insônia e estresse.^(20)^ Como o presente estudo brasileiro utilizou o questionário da WHO, que aborda sinais e sintomas psicoemocionais menos específicos, seu resultado pode gerar estimativa mais ampla do desgaste mental e, por tal motivo, é indicado para triagem nos serviços de saúde.^(14)^

O predomínio de participantes do sexo feminino na amostra é compatível com dados globais que indicam maior frequência de mulheres na força de trabalho do setor da saúde (70%).^(21)^ Os dados do presente estudo demonstraram que a chance de sofrimento mental entre as trabalhadoras brasileiras foi duas vezes maior do que entre os homens. Na Ásia, as trabalhadoras de saúde apresentaram maior frequência de sintomas depressivos (26,9% *versus* 20,3%) e ansiosos (29,1% *versus* 20,9%),^(9)^ além de quadros mais graves de transtornos mentais.^(20)^ Essa diferença entre os sexos tem diversas explicações psicossociais relativas ao mundo do trabalho, como, por exemplo, menor remuneração da mulher e ocupação de cargos menos valorizados dentro do ramo da economia dos serviços de saúde.^(21)^ A questão da dupla jornada (trabalho-casa) pode ser um fator relevante para esse resultado, considerando o risco de aumento da carga de trabalho global nesse cenário de pandemia, com a intensificação das tarefas de trabalho e domiciliares.

Os participantes com menos de 40 anos tiveram maior chance de sofrimento mental, resultado este que está alinhado a dados pré-pandêmicos.^(22,23)^ Em estudo realizado no Reino Unido durante o surto global pelo SARS-Cov-2 não houve diferença entre as faixas etárias quanto aos sintomas psicológicos,^(10)^ mas, em cenários de surtos virais prévios, os trabalhadores mais jovens e aqueles com menos experiência eram mais predispostos a apresentar distúrbios psicoemocionais.^(24)^ Uma hipótese para esse resultado seria a de que os trabalhadores com idade mais avançada e, provavelmente, com mais anos de profissão tenham desenvolvido estratégias que permitiram um enfrentamento menos traumático dos estressores pessoais e profissionais decorrentes do contexto da COVID-19.

Não foi encontrada diferença na frequência de desgaste mental entre as profissões dos participantes da pesquisa. Entretanto, revisão sistemática indicou que a equipe de enfermagem tinha maior frequência de quadros depressivos e ansiosos do que a equipe médica;^(9)^ estudo chinês demonstrou sintomas mais graves no primeiro grupo.^(20)^ Talvez a complexidade do contexto pandêmico brasileiro, com estressores coletivos do ambiente e da organização do trabalho, promova um impacto negativo equânime entre os profissionais, independentemente das questões específicas de cada uma das funções. Por exemplo, a indisponibilidade de equipamentos de proteção individual e a sobrecarga de trabalho,^(25)^ além do medo de ser contaminado, podem ser estressores psicológicos comuns e frequentes entre aqueles que estão na linha de frente do atendimento dos pacientes.^(8)^ O estudo qualitativo com profissionais de enfermagem na China demonstrou que a equipe apresentou sentimentos positivos e negativos durante a pandemia, sendo necessário o desenvolvimento de estratégias de enfrentamento individuais e coletivas para mitigar os impactos negativos do trabalho, a fim de manter seu desempenho profissional.^(7)^

A revisão de Shaukat et al.,^(6)^ relaciona o desgaste mental às condições psicossociais do trabalho de profissionais da saúde durante essa pandemia. Os dados do presente estudo brasileiro demonstram que as características psicossociais apresentaram importante associação com o sofrimento mental. A alta exigência no trabalho, com grande demanda qualiquantitativa de tarefas e baixa latitude de decisão para realizá-las, não foi a característica mais frequente entre os participantes, mas aumentou em quase 150% a chance do agravo em estudo. Na Alemanha, foi observado que o aumento na carga de trabalho e as mudanças organizacionais estiveram associados a esgotamento profissional e desgaste psicológico entre profissionais de saúde.^(8)^ Outros estudos realizados com trabalhadores da saúde antes da pandemia encontraram resultados similares quanto à associação entre estressores tóxicos do trabalho e sofrimento mental.^(16,18)^ Além dos estressores comuns ao trabalho em serviços de saúde, provavelmente há características inerentes ao sistema de saúde público brasileiro, como tensão no trabalho, redução da disponibilidade de insumos e precarização nas relações trabalhistas, que podem ter sido agravadas pelo cenário de combate à COVID-19.^(26)^

Outro exemplo dos estressores laborais é a longa jornada de trabalho. A carga horária semanal igual ou superior a 60 horas aumentou a chance de desgaste no grupo estudado. A mudança na organização de trabalho após o início da pandemia, com aumento da carga geral de trabalho, esteve associada a quadros de depressão (RC: 2,00; IC95%: 1,33-3,02) e ansiedade (RC: 2,24; IC95%: 1,50-3,36) entre trabalhadores da assistência à saúde, na China.^(27)^ Afinal, uma jornada de trabalho mais extensa, como a dos participantes deste estudo, aumenta o tempo em contato com aspectos negativos no trabalho, deteriorando a saúde física e emocional.^(28)^

Em um cenário de risco de infecção, deve ser permanente o gerenciamento da efetividade das ações que protejam os trabalhadores do agente biológico.^(6)^ Na Alemanha, os trabalhadores sugeriram ações para melhorar as condições de trabalho durante a pandemia, como adequação das equipes à demanda pelos serviços, clareza nas diretrizes de organização e planejamento e melhor comunicação das chefias com a equipe.^(8)^ A China, primeiro país acometido pela infecção, estabeleceu diretrizes^(29)^ para controlar o impacto negativo da pandemia na saúde dos trabalhadores, como o equilíbrio entre horas de trabalho e de descanso, o fortalecimento de ações de segurança e a oferta de suporte para a saúde.

A percepção de boas relações entre os colegas de trabalho e seus superiores hierárquicos pode influenciar positivamente a saúde.^(12)^ Esse apoio social é capaz de modular e balancear contextos geradores de estresse, podendo fazer com que demandas sejam experimentadas como estímulo, o que resulta em um menor “custo de realização” da tarefa a ser executada.^(30)^ Nesse sentido, como observado no presente estudo e em outras pesquisas,^(16,18)^ profissionais da saúde com percepção de baixo apoio social no trabalho apresentaram maior chance de sofrimento mental.^(16,18)^ No cenário da pandemia, com o aumento da carga física e emocional no trabalho, aliado a um limitado acesso a serviços de suporte psicológico,^(28)^ torna-se compreensível a alta chance de sofrimento mental quanto menor for o suporte dos colegas.^(8,31)^

Nesse contexto pandêmico, as recomendações chinesas incluem o apoio presencial para lidar com a carga psicológica e a oferta de serviços de telessaúde.^(29)^ Portanto, além da melhoria das condições de trabalho, há ganhos ao se organizarem equipes que ofereçam acolhimento, suporte e intervenções coletivas e individuais aos trabalhadores.^(6,31)^ Podem ser desenvolvidas estratégias para minimizar o medo diante do risco de adoecer ou de infectar conhecidos, o convívio com a dor e a morte de pacientes e colegas de trabalho e outras questões que causam desgastes, como as condições precárias do trabalho ilustradas nos resultados desta pesquisa. É importante que tais ações sejam implantadas em caráter preventivo a todos os trabalhadores e institucionalizadas enquanto política organizacional permanente de saúde e segurança no trabalho.

Considerando que este estudo é pioneiro na avaliação da saúde mental de trabalhadores e dos estressores psicossociais no trabalho durante a pandemia da COVID-19, com escalas psicométricas validadas e técnica estatística de controle de variáveis de confundimento, há limitações que devem ser levadas em consideração ao se extrapolarem seus resultados: houve a participação de indivíduos com melhor acesso às ferramentas tecnológicas e de conectividade à internet; apesar da abrangência nacional, há um descompasso entre a distribuição demográfica do país e o local de moradia dos participantes; o maior interesse pela pesquisa de pessoas com sinais e sintomas de sofrimento mental e a impossibilidade de avaliar perdas; o uso de questionários autorreferidos, que podem ser impactados pela repercussão cognitiva dos quadros clínicos dos participantes; e o delineamento transversal do estudo, que não permite inferir causalidade entre o desfecho e as covariáveis.

## CONCLUSÃO

O sofrimento mental esteve presente em seis a cada dez trabalhadores de serviços de saúde engajados no atendimento de pacientes durante a pandemia de COVID-19 que participaram do estudo. Fatores individuais influenciam no aumento de chance do desgaste mental, como sexo feminino e idade inferior a 40 anos.

As características psicossociais do trabalho tiveram forte associação com o desfecho, como a percepção do trabalho de alta exigência, a jornada semanal de trabalho igual ou superior a 60 horas e o baixo apoio dos colegas de trabalho. Assim, é urgente a necessidade de mapear os serviços de saúde que tenham essas características para delineamento de ações de promoção da saúde mental e prevenção do desgaste emocional nos diversos níveis de atenção.

Considerando o impacto dos aspectos do contexto e do conteúdo do trabalho encontrados neste grupo, é mandatória uma política nacional para avaliação e mitigação do risco psicossocial laboral. A implantação de estratégias que protejam os profissionais da saúde dos quadros psicossomáticos pode auxiliar no controle das repercussões negativas permanentes no bem-estar emocional, e na qualidade de vida dos trabalhadores.

Por fim, sugerem-se pesquisas adicionais de delineamento longitudinal e com amostras representativas de cada região do país, para aprofundar a discussão nacional sobre o impacto do trabalho na saúde do trabalhador e que possibilitem a indicação de intervenções eficazes.
